# A Review of the Dissemination of Antibiotic Resistance through Wastewater Treatment Plants: Current Situation in Sri Lanka and Future Perspectives

**DOI:** 10.3390/life14091065

**Published:** 2024-08-25

**Authors:** Wasana Gunawardana, Ruwani S. Kalupahana, Sanda A. Kottawatta, Ashoka Gamage, Othmane Merah

**Affiliations:** 1 China Sri Lanka Joint Research and Demonstration Centre for Water Technology (JRDC), E.O.E Pereira Mawatha, Meewathura Road, Peradeniya 20400, Sri Lanka; ashogamage@gmail.com; 2 Department of Veterinary Public Health and Pharmacology, Faculty of Veterinary Medicine and Animal Sciences, University of Peradeniya, Peradeniya 20400, Sri Lanka; ruwanikalupahana@yahoo.com (R.S.K.); sanda@vet.pdn.ac.lk (S.A.K.); 3 Chemical and Process Engineering, Faculty of Engineering, University of Peradeniya, Peradeniya 20400, Sri Lanka; 4 Laboratoire de Chimie Agro-Industrielle, LCA, Institut National de la Recherche Agronomique et Environnement, Université de Toulouse, 31030 Toulouse, France; 5 Département Génie Biologique, Institut Universitaire de Technologie Paul Sabatier, Université Paul Sabatier, 32000 Auch, France

**Keywords:** wastewater treatment plants, Sri Lanka, antibiotic resistance, antibiotic-resistant genes, public health risks, treated effluent, receiving water bodies

## Abstract

The emergence of antibiotic resistance (AR) poses a significant threat to both public health and aquatic ecosystems. Wastewater treatment plants (WWTPs) have been identified as potential hotspots for disseminating AR in the environment. However, only a limited number of studies have been conducted on AR dissemination through WWTPs in Sri Lanka. To address this knowledge gap in AR dissemination through WWTP operations in Sri Lanka, we critically examined the global situation of WWTPs as hotspots for transmitting antibiotic-resistant bacteria (ARB) and antibiotic-resistant genes (ARGs) by evaluating more than a hundred peer-reviewed international publications and available national publications. Our findings discuss the current state of operating WWTPs in the country and highlight the research needed in controlling AR dissemination. The results revealed that the impact of different wastewater types, such as clinical, veterinary, domestic, and industrial, on the dissemination of AR has not been extensively studied in Sri Lanka; furthermore, the effectiveness of various wastewater treatment techniques in removing ARGs requires further investigation to improve the technologies. Furthermore, existing studies have not explored deeply enough the potential public health and ecological risks posed by AR dissemination through WWTPs.

## 1. Introduction

Antimicrobial agents are essential drugs for protecting public and animal health [1,2,3,4]. However, the inappropriate use of antibiotics results in their continuous release into natural environments causing public health and ecological problems [1,5,6,7,8,9,10,11,12]. Antibacterial resistance occurs naturally over time due to the release of antibiotics into the environment [3,4,13,14]. Therefore, since the time that antibiotics were introduced, scientists have been guided on the proper use of antimicrobials, because the misuse of antimicrobials leads to antimicrobial resistance (AMR) [15,16]. In the environment, antibiotics can cause selection pressure, which eliminates sensitive microorganisms and brings about the survival of resistant cells, allowing them to overcome the adverse effects of antibiotics. At present, many pathogenic diseases causing microbes have developed resistance to commonly used antimicrobials with the emergence of multidrug-resistant (MDR), extensively drug-resistant (XDR), and pan-drug-resistant (PDR) bacteria [17,18,19,20]. The emergence of multiple drug-resistant pathogenic species in a water environment is even more problematic, particularly when surface water fulfills the urban drinking water demands [15,21].

Consequently, the infections caused by the AR superbugs become untreatable with existing antibiotics, and the World Health Organization (WHO) has published its first-ever list of “priority pathogens”, for which new antibiotics are needed [3]. In February 2017, the WHO announced that AMR is a growing process and “one of the top 10 global public health threats facing humanity” [3]. According to the data presented in the WHO’s global health estimates, about one in six deaths could be due to drug-resistant strains of tuberculosis, malaria, HIV, and bacterial infections by the year 2050. As in many developing countries, at present, spreading antibiotic resistance is an emerging issue in Sri Lanka [5,22,23,24,25]; furthermore, MDR is a significant problem in hospital settings in most countries, including Sri Lanka [26,27,28,29].

**Figure 1:**
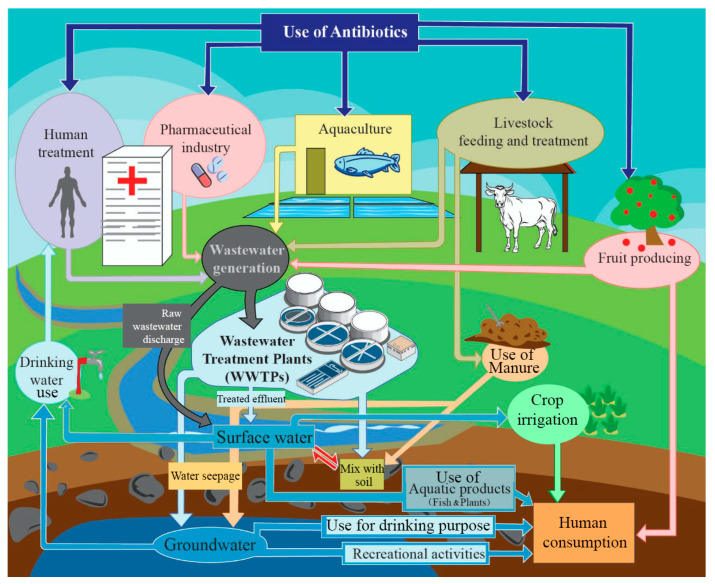
Schematic diagram showing the different pathways of transmission of antibiotics and ARGs in the environment due to the operation of WWTPs, the prevalence of AR in the environment, and human exposure to ARB and ARGs.

Wastewater treatment plants are known as “hotspots” for spreading AR in various environments [6,18,30,31,32,33,34,35,36,37]. These WWTPs mainly utilize a combination of physical, chemical, and biological processes to eliminate pollutants from wastewater [38,39,40]. Within WWTPs, a diverse group of organisms including bacteria, protozoans, metazoans, viruses, and fungi play the main role in wastewater treatment [41,42]. However, the WWTPs provide a better environment for ARB and ARGs to occur and spread [6,33,37,43,44]. The dissemination of antibiotics, ARB, and ARGs through treated effluents [7,36,45], sludge [34,46,47,48], and also aerosols [49,50] from WWTPs into the environment has been reported in many studies [24,35]. Furthermore, a wide variety of clinically important ARB has been detected in the effluent and sludge of WWTPs [51,52,53]. A detailed diagram showing the spread of AR in the environment due to WWTPs and the pathways of human exposure to AR is given in Figure 1.

It has been reported that a wide range of removal efficiency of antibiotic residuals, ARB, and ARGs can be achieved during wastewater treatment depending on the treatment processes used [54]. However, the operation of WWTPs poses challenges when they operate in the long run; WWTPs face difficulties in meeting the discharge wastewater quality standards, mostly due to insufficient process monitoring and maintenance activities, a lack of appropriate systems, and the high cost of operation, thus adversely affect the quality of the final effluent [55]. The final effluent disinfection processes such as chlorination and UV disinfection are not as efficient as expected with the poor-quality effluent [56,57]. As a result, a large number of living organisms, including pathogenic bacteria and ARB, could be released into receiving water bodies [55,58]. Among various pathogenic environmental bacteria, *Arcobacter* spp. are frequently detected in environmental samples [27]. 

Therefore, understanding the paths of the development and spread of ARB and ARGs in the environment through WWTPs is important for implementing precautionary strategies to control the process. Therefore, in this review, the authors critically examined the global situation of WWTPs as “hotspots” for transmitting ARB and ARGs to address the current situation, the potential for control, and the knowledge gap in AR dissemination through the currently operating WWTPs in Sri Lanka. 

## 2. Materials and Methods

In this review, the literature search was efficiently conducted through prominent online databases including ScienceDirect, Scopus, PubMed, and Google Scholar. For the literature search, keywords and a combination of keywords, including wastewater, wastewater treatment plants, wastewater management in Sri Lanka, types of wastewaters, wastewater characteristics, antibiotic resistance (AR), antibiotic-resistant bacteria (ARB), antibiotic-resistant genes (ARGs), treated effluent, sludge, receiving water bodies, pathogens, public health risks of AR, ecological problems of AR, pathways of spread of antibiotic resistance in the environment, ARGs in sludge and treated effluent, the prevalence of antibiotic resistance in the environment, human exposure to AR, spread of antibiotic resistance through WWTPs in Sri Lanka, were used. In addition, a thorough search was conducted on the relevant reputable websites, such as the World Health Organization (WHO), Environmental Protection Agency (EPA), and World Food Programme (WFP), using common search engines. In total, 105 pieces of literature, including prior reviewed articles, theses, technical reports, and conference papers were considered in this review. To ensure the relevance of the literature, the spread of AR only through WWTPs was included in this review. A summary of the reviewed articles is given in Table 1.

## 3. Results and Discussion

### 3.1. The Worldwide Situation of Releasing Antibiotics into the Environment through WWTPs

It is widely recognized that a significant amount of antibiotics are released into municipal wastewater through urine and feces as a result of incomplete metabolism in human and animal bodies [5,6]. Studies have shown that approximately 70% of tetracycline antibiotics are excreted and released in an active form into the environment through human and animal waste [30,37,59]. Additionally, it has been reported that 15–25% of quinolones are excreted through urine and 45–62% through feces [6,60]. Fluoroquinolones (FQs) have been found in high concentrations in sewage treatment plants in Sri Lanka [24]. These antibiotics ultimately make their way through WWTPs into the environment [9,24,44,56,61,62,63]. Moreover, the improper disposal of antimicrobials from hospitals, livestock, and communities also contributes to the contamination of the environment, particularly through wastewater and manure [27,44,64,65]. Hospital wastewater is a significant source of antibiotic dissemination in aquatic environments. Studies have shown that antibiotic concentrations in hospital effluent are often several times higher than in sewage treatment plant effluent due to the high usage and low dilution compared to household effluent [44]. 

Studies have identified the presence of various antibiotics, including tetracycline, sulfonamides, ofloxacin, azithromycin, trimethoprim, and metronidazole, in the effluent-receiving rivers [36,65]. Interestingly, higher concentrations of antibiotics have been detected in downstream water samples compared to upstream samples [17,34,39]. Further research is necessary to understand the impact of pollution levels in these water bodies on the occurrence and persistence of ARGs and ARB. Additionally, it is important to investigate how these pollutants affect the microbial ecology of the receiving water bodies. The details of the antibiotics found in WWTPs and receiving water environments are given in Table 2.

### 3.2. The Worldwide Situation of WWTPs as Potential Hotspots for Spreading ARB and ARGs 

Wastewater treatment plants are considered to be probable hotspots for the spread of AR in the environment because WWTPs offer favorable conditions for the proliferation of ARB, as well as for the horizontal transfer of ARGs among different microorganisms [24,51,59,68]. WWTPs are home to a diverse group of microorganisms, including bacteria, protozoan, metazoan, viruses, and fungi; furthermore, wastewater is rich in nutrients, creating a fertile environment for the proliferation of ARB and ARGs [35,69]. The discharge of treated effluent plays a significant role in spreading ARB and ARGs in the effluent-receiving water bodies and the environment, due to the availability of residual antibiotics [37,70,71,72,73]. Therefore, the effective management and monitoring of these facilities are very important. The details of the ARGs discovered in WWTPs and effluent-receiving water bodies are shown in Table 3. 

Antibiotic resistance genes have been identified in the final effluent and sludge of WWTPs globally. Numerous studies have indicated that these ARGs are diminishing the effectiveness of groups of antibiotics such as sulfonamide (*sul*), tetracycline (*tet*), fluoroquinolone (*qnr*), macrolide (*erm*), chloramphenicol (*cml, flo*), methicillin (*mec*), and b-lactam (*bla*) against pathogens [10,15,17,32,38,74,75]. Macrolide resistance genes, tetracycline resistance genes, and sulfonamide resistance genes have been detected in WWTPs with conventional treatment processes [43]. Several ARGs, including tetracycline resistance genes, sulfonamide resistance genes, and quinolone resistance genes, have been identified in rivers that receive the treated effluent from WWTPs [62]. The prevalence of ARGs, including tetracycline (*tetA*), beta-lactams (*blaCMY*), and sulfonamide (*sul1*), has been commonly observed in *E. coli* isolates [76,77]. Furthermore, research has shown that the total concentration of ARGs in sludge from hospital WWTPs in China was three to four times higher than in samples from residential WWTPs [25,44,56]. 

According to culture-dependent methods, the commonly detected ARB species in WWTPs include indicator organisms such as *E. coli*, coliforms, and enterococci. Additionally, molecular biology methods have revealed a diverse array of clinically important ARB in the final effluent of WWTPs [10,68,78]. Examples include methicillin-resistant *Staphylococcus aureus* (MRSA) [19,27,64], vancomycin-resistant *Enterococcus* [27], multidrug-resistant *Mycobacterium tuberculosis* [31], quinolone-resistant *E. coli*, and carbapenem-resistant Enterobacteriaceae bacteria [47,53,78]. Furthermore, *E. coli* strains resistant to cefotaxime, including extended-spectrum beta-lactamase (ESBL) producers, ciprofloxacin, and cefoxitin, have been identified in treated effluent samples [56,62]. Drug-resistant *Salmonella* species also pose a significant public health concern globally. Urban rivers impacted by treated effluent discharge were found to harbor antibiotic-resistant pathogens like *Klebsiella pneumoniae,* Acinetobacter, Enterococci, *Pseudomonas* spp., and *Shigella* spp. [9,18,60,73].

**Table 3:** Details of ARGs found in WWTPs and receiving water bodies.

Detected Resistance in Antibiotics and Antibiotic Class/Related Mutations	Detected ARGs	Source of Antibiotics Found	Literature
Quinolones	*gyrA*, *qnrS*, *gyrB*, *parC*	WWTPs and receiving water bodies and their biofilms	[9,57]
Sulfonamides	*Sul1*, *Sul2*	WWTPs	[34,60,76]
Beta-lactams	*bla*AmpC, *bla*SHV	Wastewater and WWTPs	[34,67,68]
(β-lactams)			
Macrolide	*ermB, macB, ereA*	WWTPs	[34,79]
Tetracycline	*tetA, tetO, tetW, tetM, tetG*	WWTPs and reclaimed water	[51,60]
Tetracycline	*bla*CMY	*E. coli* isolates from WWTPs	[76,79]
β-lactams sulphonamide	*Sul1*		
Sulfonamides tetracycline	*ampC*	WWTPs, wastewater, and urbanized sites of the Kelani River, Sri Lanka	[15,20,22,34,57,79]
Beta-lactams fluoroquinolones	*bla*CTX		
	*sul1, tetW*		
	*gyrA*		
	*aac-(6′)-1b-cr, qnrB, qnrS, ampC*		

### 3.3. The Nature of Antibiotic Use in Sri Lanka

In recent years, there has been a significant rise in antimicrobial consumption, particularly in low- and middle-income countries (LMICs), as these drugs have become readily available [23,80]. The use of antibiotics deemed crucial for human health increased by 91% worldwide and 165% in LMICs between 2000 and 2015 [23]. For example, seventy-one countries, including Sri Lanka, experienced a 36% surge in antibiotic consumption from 2000 to 2010. The global market for antibiotics sees an estimated annual consumption of between 100,000 and 200,000 tonnes, with approximately 50% of these antibiotics being used in veterinary medicines [12,81] and as growth promoters. There has been a noticeable shift in the top ten oral and parenteral antibiotics from narrow-spectrum to broad-spectrum options. 

Due to a lack of strict regulations, many local people use antibiotics to treat self-diagnosed illnesses or symptoms, such as the common cold, a mild sore throat, and viral diarrhea, even though the provision to purchase medicines without a prescription has been forbidden in Sri Lanka since 1986 [3,24]. In many places, antibiotics are often given without professional clinical judgment [12,82]. In addition, the poorly regulated private medical sector, high rates of hospital infections, and frequency of infectious diseases have also accelerated the use of antibiotics in developing countries like Sri Lanka [25,26,27]. According to the state sector data, antibiotic consumption increased from 6.79 DIDs (Defined Daily Doses per 1000 inhabitants) in 1994 to 13.89 DIDs in 2018, with the number of substances increasing from 19 to 41, respectively, in Sri Lanka; the most consumed drug was a beta-lactam antibacterial [11,83]. Based on the available data, the total antibacterial consumption (ABC) in 2017 amounted to 343.46 million Defined Daily Doses (DDDs), whereas the private sector accounted for 246.76 million DDDs. It has been reported that the beta-lactam penicillin antibacterial group represented 59% within the public sector, whereas it accounted for about 27% in the private sector. On the other hand, macrolides, quinolones, and other beta-lactam antibacterials made up 60% of private sector consumption compared to 28% in the public sector [84]. Furthermore, Sri Lanka attracts more tourists from all over the world (www.cbsl.gov.lk, accessed on 24 February 2022), and the tourists are more likely to use antibiotics that are rarely consumed by local people [24]. 

According to the National Strategic Plan for Combating Antimicrobial Resistance in Sri Lanka (2017–2022), the overall rates of antimicrobial resistance in the country are closely linked with antimicrobials. Compared to developed countries like the United Kingdom, Sri Lanka has significantly higher rates of resistance among organisms to antibiotics. A report from the national surveillance of antimicrobial resistance in Sri Lanka in December 2014 showed that 30% of Salmonella Typhi and 36% of Salmonella Paratyphi are resistant to ciprofloxacin [27]. Furthermore, a concerning level of resistance to meropenem was detected in the Acinetobacter species isolated from patients in intensive care units in Sri Lanka [85].

The national surveillance on urinary tract infections also highlighted a troubling trend. Coliforms showed a high resistance rate to cefotaxime and ciprofloxacin. These findings underscore the urgent need for comprehensive strategies to address antimicrobial resistance in Sri Lanka.

### 3.4. Current Situation of Operating WWTPs in Sri Lanka 

Currently, in Sri Lanka, there is a wide array of industries, commercial facilities, hospitals, livestock farms, hotels, Export Processing Zones (EPZs), and housing schemes that rely on onsite (decentralized) WWTPs [86]. While a few centralized WWTPs are operating, ongoing projects are primarily focused on implementing centralized treatment technologies in urban areas of the country. Typically, wastewater is treated using a combination of primary and secondary treatments (biological or chemical) with or without tertiary treatments [69]. Despite the numerous advantages, operating WWTPs can pose challenges in the long term, primarily due to a lack of appropriate wastewater treatment technologies and insufficient skills in process monitoring and maintenance. Consequently, issues related to the discharge quality of treated effluent, sludge management, treated water, and sludge reuse, as well as a lack of knowledge and awareness, are among the main challenges faced by WWTP operators. Many operational Onsite Wastewater Treatment Plants (OWTPs) struggle to meet the wastewater quality standards outlined in the National Environmental Act of Sri Lanka [86]. 

In contrast, the effective functioning of conventional biological processes relies heavily on gravimetric settling for the separation of treated effluent from sludge (biomass) [69]. However, achieving a proper separation of the biomass is often hindered by various issues within the microbial component of the biological processes [41,87]. Problems such as filamentous bulking, foaming, pin-flocs, and the dispersed growth of bacteria pose challenges in separating the biomass from the treated water, consequently impacting the quality of the final effluent and the overall efficiency of the treatment process [55,69]. Moreover, instances of sludge rising, where the biomass floats on the surface of the clarifier tank, are commonly observed in temperate countries like Sri Lanka, particularly on sunny days with high temperatures due to the proliferation of denitrifying bacteria. This phenomenon leads to the discharge of significant amounts of suspended particles (biomass) in the final effluent, containing living bacteria and cell debris. In Sri Lanka, the treated effluent from the WWTPs is discharged into surface water bodies such as rivers, lakes, and ponds. This water is then directly or indirectly reused for both potable and non-potable purposes by the local population [21,36,39,69,88,89]. Therefore, ensuring the quality of the final effluent is crucial in preventing the spread of ARB and ARGs through treated effluents into the surrounding environment. Further research is imperative to comprehend the impact of microbial issues, such as sludge rising, and temporal dynamics on the dissemination of ARB and ARGs in the environment.

Tertiary treatment processes such as chlorination and UV disinfection are commonly used to disinfect the final effluent before it is discharged into the environment [90]. However, if the final effluent contains a significant amount of suspended particles, the disinfection process may not be as effective as anticipated by the plant operators [86]. Additionally, the monitoring of indicator organisms in the treated effluent before discharge is not consistently conducted, resulting in the release of a large number of living organisms, including pathogenic bacteria, ARB, and ARGs, into effluent-receiving water bodies through the discharge of the final effluent and sludge [33,46,91,92]. The surface water in the receiving water bodies is utilized directly or indirectly by the downstream communities for both potable and non-potable purposes. Consequently, ARB have the potential to circulate within populations of humans and animals through contaminated food and water sources [51]. Recent studies have explored the seasonal variation of antibiotic resistance in rivers in both Sri Lanka and India [15,24,93]. Therefore, proper monitoring and disinfection protocols must be implemented to mitigate the spread of harmful organisms and genes in effluent-receiving water bodies.

### 3.5. Antibiotics, ARB, and ARGs Identified in WWTPs and Receiving Water Bodies in Sri Lanka 

The occurrence, consumption, and removal efficiency of antibiotics in WWTPs handling domestic wastewater from both local and tourist communities have been investigated and antibiotic agents such as sulfamethoxazole, trimethoprim, norfloxacin, ofloxacin, and chlortetracycline have been found in all the effluent samples analyzed [24,93,94]. In pioneering research conducted in Sri Lanka in March 2018, a comparison was made between the effluents and influents from two WWTPs located in communities frequented by tourists and Sri Lankan residents. The study revealed that the tourists were consuming a higher daily dose of antibiotics (0.25 g/1000 persons) compared to the residents. In particular, it was observed that the tourists in the area under study appeared to be using more antibiotics during the research period, with an estimated daily dose of 0.25g per 1000 persons. Interestingly, azithromycin (AZM) has been identified as the most dominant antibacterial agent found in primarily treated wastewater, and azithromycin (AZM) is not commonly used by local communities in the study area [24]. Furthermore, the prevalence of ARB, ARGs, and MDR was detected in two municipal WWTPs treating wastewater from hospital and municipal areas in the western and southern regions of Sri Lanka; upon comparison with the global data, it was revealed that the WWTPs in Sri Lanka exhibit higher levels of antibiotic resistance compared to those in India. Notably, *E. coli* strains from all sampled locations in both Sri Lanka and India displayed multidrug resistance, highlighting a significant public health concern. 

Rivers serve as a medium for the proliferation and dissemination of AR among bacteria as evidenced by various studies [15,75]. Studies have highlighted the correlation between the rise of ARGs and ARB with elevated levels of antibiotics, nutrients, metals, and microplastic contaminants in effluent-receiving water bodies [92]. A recent investigation conducted by Manish Kumar et al. (2020) explored the presence of ARB, ARGs, and metal concentrations in the Kelani River of Sri Lanka, aiming to comprehend the seasonal variations in their prevalence. Tetracycline and sulfamethoxazole emerged as the most resistant antibiotics in the Kelani River in Sri Lanka; the Kelani River serves as a vital source of drinking water for the Sri Lankan population. In urbanized sections of the Kelani River, ARB and ARGs exhibited resistance to a range of antibiotics, including norfloxacin, ciprofloxacin, levofloxacin, kanamycin monosulfate, tetracycline, and sulfamethoxazole. Furthermore, the study revealed a higher resistance rate to older antibiotics like tetracycline and sulfamethoxazole compared to newer antibiotics [15,22]. 

Furthermore, research has been conducted on the prevalence of AR in tropical rivers including the Kelani and Gin Rivers in Sri Lanka, as well as the Sabarmati and Brahmaputra Rivers in India. The findings revealed that nearly all sampling points in both countries contained E. coli strains that exhibited resistance to more than one antibiotic. A higher prevalence of resistance to E. coli was observed in municipal canals in Sri Lanka compared to the Gin and Kelani Rivers, attributed to lower flow rates. Additionally, a study reported a higher incidence of AR in the surface water in Guwahati, India, compared to the Gin and Kelani Rivers in Sri Lanka [22]. Remarkably, resistant *E. coli* isolates for tetracycline and sulfamethoxazole, as well as resistant genes for fluoroquinolones (aac-(6′)-Ib-cr, *qnrB*, *qnrS*), β-lactams (*ampC*), and sulfonamides (*sul1*), were detected in samples from the Kelani River in Sri Lanka. As India is a hotspot for using antibiotics and the emergence of new antibiotic resistance phenotypes and Indian and Sri Lankan people also have close contact, the urgent need for further research and interventions to address the growing issue of antibiotic resistance in both countries is highlighted [22]. 

The improper disposal of sludge from WWTPs is a significant environmental concern [31,48]. However, there is a lack of research in Sri Lanka on the factors contributing to the emergence and spread of antibiotic-resistant bacteria (ARB) through WWTP sludge. The studies conducted worldwide have indicated the potential for pathogenic organisms, antibiotics, ARB, and ARGs to be discharged into water environments through WWTP sludge [35]. Additionally, it has been reported that sludge can become contaminated with approximately 300 species of bacteria, with bacteria able to survive in the environment for several months [44,48]. Therefore, further investigations into the transmission of AR through sludge are necessary. Research has also shown a correlation between ARGs and the concentrations of antibiotics present in sludge [7,38]. 

### 3.6. The Severity of the Situation of AR Spread through WWTPs in Sri Lanka 

Concerns regarding AR in Sri Lanka are highlighted due to the absence of a functioning official surveillance system. Despite the presence of an officially launched strategic plan to control AMR, Sri Lanka still lacks a comprehensive monitoring mechanism [83]. Antibiotic resistance is a key challenge faced by the healthcare sector of Sri Lanka for the past few years, as antibiotic resistance has been a pressing issue for several years [27]. Recent data indicated that over 12% of the total estimated healthcare budget (around LKR 3.3 billion) in Sri Lanka is allocated to antimicrobial medicine, which poses challenges in treatment and a financial burden in patient management; as a result, the rising costs associated with antibiotic resistance would be unfavorable for providing cost-effective healthcare facilities in the future.

In December 2014, the national surveillance report on antimicrobial resistance, submitted by the Sri Lanka Medical Council (SLCM) to the Ministry of Health (MoH), revealed a concerning trend of previously antibiotic-sensitive organisms developing resistance. Along the given examples, enteric fever, which encompasses typhoid and paratyphoid fevers caused by *Salmonella Typhi* (*S. Typhi*) and *Salmonella Paratyphi* (A, B, and C), is prevalent in many developing countries, including Sri Lanka [85]. Of particular concern is the emergence of resistance in commonly used ciprofloxacin antibiotics to *S. Typhi* and *S. Paratyphi* A in Sri Lanka. *Salmonella Paratyphi* A, in particular, has become a common cause of enteric fever in adults, with a staggering 92% resistance to ciprofloxacin [85]. According to the weekly epidemiological reports of the Epidemiological Unit of Sri Lanka, approximately 1300–3000 clinically suspected cases of enteric fever are reported each year, and there is a noticeable shift from typhoid fever to paratyphoid fever, with *S. Paratyphi* A emerging as the predominant causative agent in certain provinces. Further studies are required to confirm this shift from typhoid fever to paratyphoid fever and its implications [85]. It is worth noting that the majority of isolates were resistant to ciprofloxacin and are showing emerging resistance to azithromycin, as reported by Suchetha Charukeshi Illapperuma et al. (2019) [95]. This underscores the urgent need for continued surveillance and research to address the growing threat of antimicrobial resistance in Sri Lanka. 

Furthermore, Acinetobacter species isolated from patients in intensive care units have exhibited a significant level of resistance to meropenem. Studies have also indicated that the Acinetobacter species responsible for Ventilator-Associated Pneumonia (VAP) are multidrug-resistant, with resistance rates of 90% to cefotaxime, 73% to ceftazidime, 70% to imipenem, and 53% to cefoperazone–sulbactam [96]. Additionally, the national surveillance on urinary tract infections has shown a high resistance rate in coliforms to broad-spectrum antibiotics such as cefotaxime and ciprofloxacin [97]. Coliforms are the most common group of organisms responsible for urinary tract infections [26], with hospitalized patients, particularly in the pediatric age group, exhibiting higher resistance rates compared to outpatients. Notably, high resistance rates in coliforms were observed for orally available antibiotics, except for nitrofurantoin, even in pediatric practices. Between 2014 and 2016, the majority of coliform isolates displayed very high resistance rates to ampicillin (85%) and moderate resistance to cefalexin (44.8%), cefotaxime (36.6%), co-amoxiclav (36.3%), ciprofloxacin (46.2%), and gentamicin (23%) [11]. Methicillin-resistant *Staphylococcus aureus* is a significant nosocomial pathogen in Sri Lanka, causing a range of infections from mild to life-threatening. The prevalence of MRSA in Sri Lanka varies across different hospital settings [27,29]. 

On the contrary, infections caused by ARB are associated with increased mortality and morbidity rates, as well as prolonged hospital stays [28]; this poses a significant health challenge for developing countries like Sri Lanka, where a high prevalence of infectious diseases is observed [98]. Although the country has made improvements in social and economic progress, a considerable portion of the population continues to live with poor sanitation and housing conditions, which accelerate the person-to-person and environmental spread of resistant pathogens and genes [3]. Additionally, the rise of AR has substantial economic consequences, as second and third-line drugs are significantly more costly than first-line drugs. This high cost could lead to treatment failure in many individuals [99].

### 3.7. Impacts of Different Treatment Processes on Removing ARB and ARGs 

Primary and secondary treatment methods are commonly used in the conventional WWTPs used for sewage treatment. The secondary treatment methods are used to reduce biodegradable organic compounds and most of the pathogenic microorganisms in wastewater [69]. In addition to secondary wastewater treatment processes, the application of advanced wastewater treatment processes is necessary to eliminate microbial contaminants [38]. Even though the removal capacity of pathogens and other bacteria is high in conventional biological WWTPs, the removal capacity of all ARB and ARGs was not the same [5,15,22,46]. The removal efficiency of ARGs depends on the type of genes present in the wastewater and the treatment processes used [31,91,100]. In addition, it has been reported that WWTPs exhibit more consistent increases in AR after the treatment process [15,93]. A higher prevalence of ARB, ARGs, and MDR has been reported in some WWTPs in Sri Lankan hospitals [22]. Thus, the adequate treatment of sewage becomes critical for stopping the occurrence of MDR [25]. Compared to the influent, higher concentrations of twelve ARGs, including *tetA, tetB*, *tetE, tetG, tetH, tetS, tetT, tetX*, *sul1, sul2, qnrB,* and *ermC*, in the effluent have been observed, indicating the overall proliferation of resistant bacteria after treatment [47,101]. 

Although UV irradiation is widely used for the disinfection of the final effluent, studies have shown that the presence of ARGs in the treated effluent could potentially lead to the transfer of ARGs to the environment [54,102]. Various studies have examined the different responses of ARB and ARGs to the UV disinfection process, and thus differences in ARB and ARG reduction during UV treatment have been observed [38,43,103]. Research has indicated a positive relationship between the inactivation of ARGs and the dosage of chlorine, as well as the contact time of the disinfection process; ARGs such as sulI, tetX, tetG, and int1 are affected by these factors [68]. The studies focusing on erythromycin and tetracycline resistance genes in treated effluent revealed that chlorination disinfection may not effectively eliminate ARGs [36,57]. It has been reported that UV disinfection can be affected by organic and inorganic matter in wastewater, which could potentially reduce the efficiency of ARG removal in full-scale WWTPs [7,39]. Furthermore, the differences in the sensitivity of opportunistic bacteria, such as Enterococci, *Pseudomonas aeruginosa*, Staphylococci, and Enterobacteria, to UV disinfection have been observed [53,104]. However, further investigations are required to fill the knowledge gaps in ARB and ARG removal during UV disinfection and chlorination processes [54].

Recent studies have demonstrated that the Membrane Bioreactor (MBR) facility has shown the highest removal efficiency for a majority of ARGs, total bacteria, and ARB [7,66]. Additionally, the activated sludge process has exhibited the superior elimination of antibiotics [24]. Furthermore, anaerobic–aerobic treatment reactors, in combination with constructed wetlands and disinfection processes, have demonstrated favorable removal efficiencies [24,105]. Emerging strategies such as the use of nanomaterials and biochar in conjunction with other treatment methods are being explored for the removal of ARB and ARGs, but further investigation and research are needed to determine the efficacy of these innovative techniques [38]. Despite these advancements, the impact of various wastewater characteristics, including clinical, veterinary, community, and industrial wastewater, as well as operating conditions and the effectiveness of different wastewater treatment technologies, on the removal of ARGs has not been extensively studied in Sri Lanka [44].

### 3.8. Potential for Controlling the Spread of Antibiotic Resistance through WWTPs in Sri Lanka

In general, multisectoral collaboration and governance, expanding awareness of AR targeting different sectors and population groups, and training for students and working professionals are important for controlling the spread of AR. In September 2016, global leaders including Sri Lanka committed to combat the spread of AMR at the United Nations General Assembly in New York. The Ministry of Health in Sri Lanka has taken the initiative to develop the National Strategic Plan (NSP) for combating AMR in collaboration with the Sri Lanka College of Microbiologists (SLCM) under the “One Health Concept”, in line with the global action plan against antimicrobial resistance (GAP-AMR), which called on all countries to adopt national strategies [106]. For the first time, a National Strategic Plan for Sri Lanka was developed under the World Health Assembly, which endorsed the Global Action Plan (GAP) on AMR related to the one health concept that represents humans, animals, and agriculture as a closely linked system, in 2017, and it provided the roadmap to combat AMR from 2017 to 2022. The NSP 2017–2022 developed five key strategies to strengthen the knowledge and evidence base through surveillance and research [4,98]. In 2023, the progress in implementing the NSP 2017–2022 was evaluated with the assistance of the WHO to develop the revised and updated NSP 2023–2028 for combating antimicrobial resistance and ensuring the effective management of the health, animal, plant, food safety, and environmental sectors in Sri Lanka until 2028 (NAP-AMR 2023–2028) [107].

Even though the initiation of the NSP was the result of globally coming together, challenges persisted as national health priorities were influenced by different circumstances. Another important aspect is the need to reduce the consumption of antibiotics. People should be aware that the appropriate management of medical waste, vaccinations, or hygiene is also necessary to restrict the spread of infection. The community-level education and motivation of both medical sector employees and patients should be performed. 

Furthermore, implementing strategies for preventing the occurrence and transmission of AR organisms through WWTPs is vital. While conventional disinfectants and advanced treatment processes have been shown to disrupt bacterial cells, they often fall short of eliminating or significantly reducing the levels of ARGs [108]. Furthermore, individuals need to recognize that certain wastewater treatment methods may enhance the antibiotic resistance of the surviving bacteria, creating reservoirs for the transmission of AR to opportunistic pathogens if the treatment processes are ineffective. Efforts must be intensified to develop solutions for controlling the spread of antibiotic resistance. Establishing wastewater treatment facilities equipped with effective disinfection and sludge management practices is crucial in reducing antibiotic concentrations in the water environment.

The literature indicates that conventional wastewater management practices are not effective in the complete removal of antibiotics, ARB, and ARGs, and thus their discharges have a large potential to affect public health and aquatic ecosystems. Therefore, increasing treatment efficiency is very important. The upgrading of WWTPs with additional advanced treatments is a reasonable solution for increasing the efficiency of conventional wastewater treatment processes. The increase in efficiency will be attributed to the removal of suspended solids (biomass) in treated effluent, which increases the bacterial removal efficiency of tertiary treatment methods such as UV treatment or chlorination [108]. The modifications of ozonation or UV treatments, including the use of higher ozone concentrations/contact time or process combinations, are needed to increase the bactericidal effect and to improve the ARG removal efficiency. 

To minimize the spread of pathogenic ARB in the environment, it is crucial to effectively manage hospital wastewater. Additionally, it is also important to implement strategies aimed at optimizing the use of antibacterial compounds in medicine. While vaccines against numerous infectious agents are readily available, inadequate vaccination coverage, in conjunction with poor water and sanitation practices, leaves many individuals susceptible to infections and reliant on antibiotics for treatment. The impact of ARB can be mitigated by investing in expanding vaccine coverage, enhancing water and sanitation infrastructure, and enforcing antimicrobial stewardship protocols in healthcare facilities. The emergence and dissemination of ARB and ARGs in the environment through WWTPs may be underestimated if the unique characteristics of the wastewater (urban or hospital), types of treatment systems, size of upstream catchments, operational conditions, and solids concentration in effluent are not taken into account [56].

## 4. Conclusions

Numerous international studies have confirmed that wastewater and WWTPs are significant sources of discharging antibiotic residues and disseminating antibiotic resistance in receiving water bodies. However, research on the spread of ARGs and ARB through WWTPs in Sri Lanka is limited. The impact of various wastewater characteristics, such as clinical, veterinary, domestic, and industrial, on the removal of ARGs and the efficiency of different wastewater treatment technologies on the removal of ARGs have not been extensively studied in Sri Lanka. The existing literature indicates that not all WWTPs are equally effective in removing ARGs, with the removal efficiency varying based on the types of genes present and the treatment processes employed. Studies on the dissemination of ARGs and ARB through the WWTP sludge and its impact on the microbial ecosystems of receiving water bodies are lacking in Sri Lanka, underscoring the necessity for additional research in the area. The effective management of hospital wastewater and the prevention of ARGs spread through such wastewater are crucial considerations. Developing and implementing new technological solutions to enhance wastewater treatment efficiency, particularly in eliminating microbial contaminants, is essential in the current scenario. Additionally, understanding the temporal dynamics of ARB and ARG dissemination into the receiving water bodies and soil is needed. Further research to evaluate the impact of microbial problems, such as sludge rising and denitrification, and biomass discharge with the final effluent is important. Moreover, with the increasing practice of reusing treated effluent and sludge in the country, it is important to establish regulations for their use in agriculture and recreational activities to mitigate the spread of antibiotic resistance. Additionally, the potential risks of spreading AR to human health and ecological systems have not yet been thoroughly investigated in the country, highlighting the need for further research to address these knowledge gaps.

## Figures and Tables

**Table 1:** Summary of the reviewed articles.

Total number of articles reviewed under the different themes	108
The nature of antibiotic use	25
WWTPs as potential hotspots for spreading ARB and ARGs	35
The current situation of operating WWTPs in Sri Lanka and the spread of AR	12
Antibiotics, ARB, and ARGs identified in WWTPs and receiving water bodies	15
Influence of different treatment processes on removing ARB and ARGs	10
Controlling the dissemination of AR through WWTPs	11

**Table 2:** Antibiotic residuals found in WWTPs and receiving water environments.

Detected Antibiotics/Antibiotic Class	Source of Antibiotics Found	Literature
Quinolones (Norfloxacin, Ciprofloxacin, Norfloxacin, and Ofloxacin)	Influent and final effluent of WWTPs	[36,59,62]
Sulfonamides (Sulfamethoxazole, Sulfathiazole, Sulfasalazine, Sulfapyridine, and Trimethoprim)	Influent and final effluent of WWTPs	[6,59,61,66]
Sulfonamides	Surface water	[6,9,22,60,67]
Beta-lactams (Amoxicillin, Cloxacillin, Cefalexin, and Cefaclor)	Influent and final effluent of WWTPs	[4,9,37]
Macrolides (Erythromycin, Tylosin, Roxithromycin, Azithromycin, and Clarithromycin)	Influent and final effluent of WWTPs	[4,24,59]
Tetracycline (Oxytetracycline and Chlortetracycline)	Influent and final effluent of WWTPs	[43,61]
Sulfamethoxazole, Trimethoprim, Norfloxacin, Ofloxacin, Chlorotetracycine, and Azithromycin	The final effluent from WWTPs	[24,36]
Sulfamethoxazole, Trimethoprim, Ciprofloxacin, Tetracycline, and Clindamycin	The final effluent from WWTPs	[15,22,27,61]
Cefotaxime, Ciprofloxacin, and Cefoxitin	The final effluent from WWTPs	[56,62]
Sulfamethoxazole, Trimethoprim, Norfloxacin, Ofloxacin, and Chlorotetracycine	The final effluent from WWTPs	[6,24,59]
Fluoroquinolone Antibacterials	Hospital wastewater	[9,44,62]

## Data Availability

No new data were created or analyzed in this study.
